# Characterization of all second-order nonlinear-optical coefficients of organic *N*-benzyl-2-methyl-4-nitroaniline crystal

**DOI:** 10.1038/s41598-019-50951-1

**Published:** 2019-10-16

**Authors:** Takashi Notake, Masahiro Takeda, Shuji Okada, Takuya Hosobata, Yutaka Yamagata, Hiroaki Minamide

**Affiliations:** 1 Teraphotonics Team, RIKEN, Sendai, Japan; 2 Advanced Manufacturing Support Team, RIKEN, Wako, Japan; 3 Ultrahigh Precision Optics Technology Team, RIKEN, Wako, Japan; 4 Graduate School of Organic Materials Science, Yamagata Univ., Yonezawa, Japan

**Keywords:** Optical materials and structures, Materials for optics

## Abstract

Full elements of second-order nonlinear optical (NLO) tensor can be completely characterized for an organic NLO crystal for the first time. As-grown bulk N-benzyl-2-methyl-4-nitroaniline (BNA) crystal was processed to expose (100) and (010) crystal orientations with fine optical surfaces by using precision lathe and diamond blade. Then, every five nonvanishing second-order NLO coefficient of BNA can be determined quantitatively using the precisely processed crystals based on 1st-kind Maker fringe measurements. Our method makes it possible to clarify uncertain NLO property of any organic materials and to accelerate application study via precise device fabrications even for fragile organic materials.

## Introduction

Nonlinear optical (NLO) effects in materials have been widely utilized for optical signal-processing devices in information-telecommunication system. Organic NLO materials can be particularly expected to support these devices in next-generation highly-advanced information society, where further improvements in speed of signal-processing, frequency-bandwidth, power consumption, compactness of devices are strongly required. Gigantic nonlinearity, ultra-fast response due to π-electrons, low refractive index and its wavelength-dispersion in organic NLO materials can give breakthrough in these devices. Therefore, research and development of organic NLO materials has been becoming more and more important to establish future society.

Organic NLO crystals, such as 4-dimethylamino-N-methyl-4-stilbazolium-tosylate (DAST)^1,2^, N-benzyl-2-methyl-4-nitroaniline (BNA)^3,4^, 4-N,N-dimethylamino-4-N-methylstilbazolium 2,4,6-trimethylbenzenesulfo-nate (DSTMS)^5,6^, 2-(3-(4-hydroxystyryl)-5,5-dimethylcyclohex-2-enylidene)malononitrile (OH1)^7,8^, and 2-(4-hydroxy-3-methoxystyryl)-1-methylquinolinium 2,4,6-trimethylbenzenesulfonate (HMQ-TMS)^9,10^ have studied for mainly THz-wave generations and detections using second-order NLO effects^11–15^; however, their applications are still quite limited. To expand the use of organic NLO crystals in various fields of photonic technology, where inorganic crystals are exclusively used, the challenges in high-quality crystal growth and precise processing for bulk organic crystals must be addressed. Recently, however, techniques for growing high-quality, large-size organic NLO single crystals have been improved. For example, large size DAST and BNA single crystals, i.e., exceeding 1000 mm^3^ volume, have been successfully grown by optimizing the kind of solvents or concentration in the crystal-growing solution^16,17^. The internal crystal quality for organic DAST crystals can also be remedied by employing an annealing aftertreatment^18^. Hence, crystal growth techniques for organic NLO crystals have been advancing steadily.

On the contrary, precise processing technologies for organic crystals have hardly been developed. Since organic materials are very brittle, having hygroscopic properties, low melting temperatures and particular cleavage planes owing to weak intermolecular bonding, conventional processing technologies used for inorganic materials cannot be extrapolated to obtain arbitrary shape crystals and fine optical surfaces. Consequently, as-grown organic crystals with routine crystal habit have been used for past experiments and hence, application uses of organic NLO crystals have been limited. In order to develop novel organic NLO devices, it is necessary to precisely cut out properly shaped crystals with a particular crystal-facet from the as-grown bulk crystal. First processing for the organic crystals was reported by B. A. Fuchs *et al*.^19^. They tried single point diamond turning for organic L-arginine phosphate (LAP) crystals to generate high quality optical surfaces and achieved ten-nm level rms roughness typically. They found that there is no relation between processed surface roughness and processed crystal orientations. Then, Y. Namba *et al*., also processed organic DAST crystals using diamond turning^20^. By optimizing several conditions for processing, sub-nm rms surface roughness in 1 mm square area were obtained on the b-plane. Contrastively, they pointed out a relation between processed surface roughness and crystal orientations. In this way, technology to precisely process fragile organic materials has not been standardized yet and didn’t result in actual application of organic NLO crystals. Besides, since LAP and DAST crystals have strong ionic bonding, processing is relatively easy compared to normal organic crystals bonded by only weak intermolecular forces. Therefore, processing for normal organic crystal such as BNA is still challenging and must be optimized one by one.

Another critical problem impeding the use of organic NLO crystals is the fact that many elements of their second-order NLO tensor remain still unknown. In principle, organic NLO crystals are designed so that single molecules possess large hyperpolarizability, β. Established strategies for enhancing β often include prolonging conjugated chains to generate a π-electron pool as well as introducing donors and acceptors between conjugated chains. However, such molecules tend to have large dipole moments and centrosymmetric crystal structures are mainly obtained. Non-centrosymmetric crystals are generally obtained when some partial molecular structures, such as bulky, hydrogen-bonding and/or chiral substituents, are effectively introduced. Since these organic NLO crystals have poor crystal symmetry, there are many nonvanishing elements in the second-order NLO tensor of the crystals. Nevertheless, complete characterizations of all second-order NLO coefficients have not been attained yet for any organic NLO crystals. For example, although organic DAST crystals have a total of ten nonvanishing elements in its second-order NLO tensor, only elements d_11_, d_12_, and d_26_ have been characterized^21–23^. Since conventional as-grown DAST crystal has a routine morphology possessing dominant (001) facets, in which a- and b-axes lie, only d_11_, d_12_, and d_26_ can be measured as long as one use as-grown DAST crystals. In other words, one cannot attempt to measure every second-order NLO tensor without proper crystal processing. Even in reported values for d_11_ of DAST, there is big disagreement reaching 200%^21–23^. As for organic DSTMS and OH1 crystals, some second-order NLO tensors have been characterized using the Maker fringe technique^24,25^. However, since as-grown crystals without proper processing were used in the measurements, many elements of the second-order NLO tensors are still untouched. None of the second-order NLO tensors have been characterized yet for the organic HMQ-TMS crystal, although the β of a single molecule has been estimated^9^. In the case of the BNA crystal, M. Fujiwara *et al*. were able to measure the magnitudes of d_33_, d_32_, and d_31_ via the Maker fringe technique^26^. However, there are nonnegligible discrepancies between the experimental Maker fringe data and theoretical fitting lines, which is likely attributed to imperfect crystal processing. In this way, magnitudes of second-order NLO tensors of organic NLO crystals remain ambiguous and there is no organic NLO crystal for which all second-order NLO coefficients are characterized. The complete characterization of them mandates the precise processing for fragile organic NLO crystals.

Herein, we report a precise processing technology for the organic NLO BNA crystal at the beginning. Then, we report characterization of all elements of second-order NLO tensor for the BNA crystal using precisely processed plane-parallel (100)- and (010)-slab crystals. This is the first accomplishment of measuring all elements of second-order NLO tensor completely for organic crystals.

## Results

### Ultra-precise processing using diamond blade for organic materials

There are several methods to evaluate second-order NLO properties of organic crystals. The powder method^27^ is a qualitative or semiquantitative one, which is useful only for screening to find NLO-active crystals. Electric-field-induced second-harmonic generation^28^ and hyper-Rayleigh scattering^29^ can clarify β of molecules. However, in order to estimate second-order-NLO tensor, calculation according to the oriented-gas model^30^ using the known crystal structure is necessary. Meanwhile, the Maker fringe technique including the rotation and wedge methods is the direct way to measure each element of the NLO tensor without using phase-matched conditions. This technique was first reported by Jerphagnon *et al*., for isotropic and uniaxial crystals^31^. The technique has been improved for biaxial crystals, where almost all organic NLO crystals are categorized^32,33^. The Maker fringe technique is based on the measurement of generated second harmonic fringe patterns caused by the perpendicular rotation of a plane-parallel crystals with respect to the pump beam axis^34^. Since coherence lengths of NLO crystals under non phase-matched conditions are typically less than several µm, thin plane-parallel crystal with smooth optical surfaces, which have roughness in the order of nm, should be prepared to obtain a decent Maker fringe pattern. The top and back surfaces of the thin plane crystal should be parallel enough to guarantee the same optical path length in the crystal for both edges of the pump beam. However, conventional technologies such as polishing using abrasive particles cannot satisfy the level of precision necessary for fragile organic NLO crystals because frictional heat melts the surface and abrasive particles are embedded into the surface. To overcome this shortcoming, we applied an ultrahigh-precision cutting process by employing a precisely-controlled lathe and a diamond blade^19,20^ to organic as-grown BNA crystals. Because diamond has excellent thermal conductivity and dissipation, diamond cutting can avoid thermal damages on the surface and obtain high-grade optical surfaces. Cutting is one of the process to transfer the diamond-edge shape manufactured with extremely high smoothness and accuracy to target object. Its smoothness and accuracy can be directly transferred to cutting plane of organic crystals. Hence, ultra-precisely processing can be highly expected even for brittle organic crystals.

**Figure 1:**
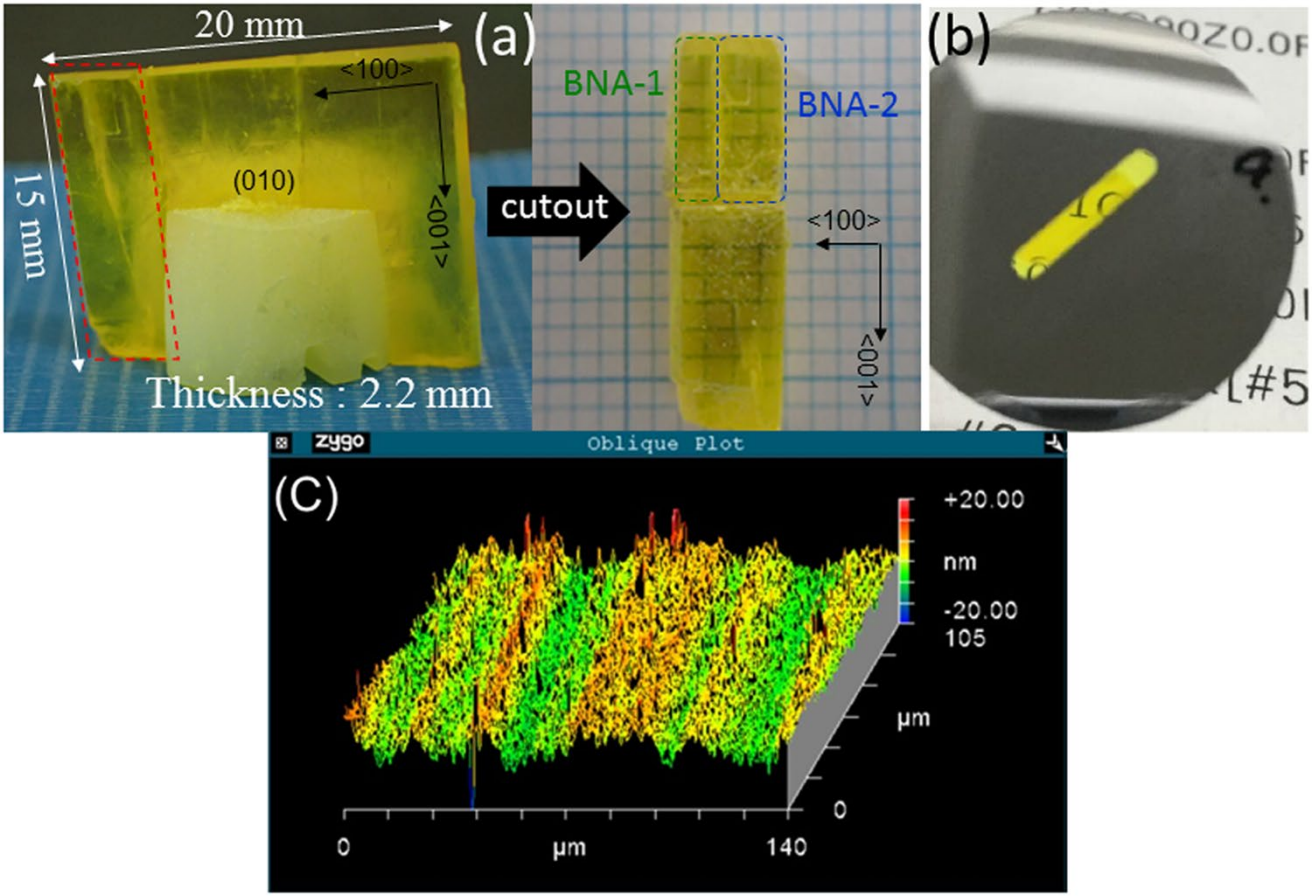
Ultra-precisely processd plane-parallel BNA crystals for Maker fringe measurments. (**a**) As-grown bulk BNA single crystal grown from solution. To make two plane-parallel BNA slab samples, certain region (surrounded by red dot-line) was roughly cutout from the bulk crystal using wire-saw firstly. Then it was devided into two pieces (BNA-1 and BNA-2) further as shown in the picture. (**b**) Photograph of processed (100)-BNA slab made from the BNA-1. In this case, both sides of (100)-plane of the BNA-1 sample were processed. (**c**) Example of measured surface profile of ceratin region of surface of the (100)-BNA slab.

BNA usually crystallizes in point group mm2 of the orthorhombic system, which possesses five nonvanishing second-order NLO tensor elements, namely d_33_, d_32_, d_31_, d_24_, and d_15_. In this case, first- and second-kind Maker fringes are expected using (100)-, (010)- and (001)-BNA slabs. In general, first-kind Maker fringe gives less discrepancy between theory and experimental data^35^. Using two plane-parallel (100)- and (010)-BNA slabs, first-kind Maker fringe patterns for d_32_, d_24_, d_33_ and d_15_, d_31_, d_33_ can be measured, respectively. Under the Kleinman symmetry law^36^, d_31_ = d_15_ and d_32_ = d_24_ can be approved for NLO crystals belong to point group mm2. However, near the band-gap region, Kleinman symmetry is no longer validated. Therefore, each of the five second-order NLO tensors of BNA should be measured independently when the pump wavelength is 1064 nm. Moreover, since coherence lengths under non phase-matched conditions are typically very short, the thickness of the plane-parallel slab sample for the Maker fringe measurement should be very thin so that neighboring fringes can be clearly observed. Because of the hexagonal cylindrical shape of as-grown BNA crystals with the largest (010) facet in which <100> and <001> axes lie orthogonally, (100)- and (010)-BNA slabs must be made by cutting as-grown bulk BNA crystals perpendicularly to the principle <100> and <010> axes, respectively. To exclude differences of individual crystal quality in the determination of elements of the second-order NLO tensor, samples to make (100)- and (010)-BNA slabs were cutout from the same as-grown BNA single crystal using wire-saw firstly, as shown in Fig. 1(a). Then, thin plane-parallel (100)- and (010)-BNA slabs were precisely processed using ultrahigh-precision cutting. A photograph of processed (100)-BNA slab is shown in Fig. 1(b). The thickness of the precisely processed (100)- and (010)-BNA slabs are 303 and 305 µm, respectively. The roughnesses of their processed surfaces were measured using a 3D optical profiler, Zygo, which has pm-level displacement resolution. Figure 1(c) shows an example of measured profile of a processed (100)-BNA slab surface, and the variance of the surface roughness was several nm. This level of surface roughness on the sample is enough to obtain a decent Maker fringe pattern and similar levels of surface roughness can also be obtained for the (010)-BNA slabs. It is important to note that relations among processed surface roughness, cut direction, cut crystal plane, cleavage directions, rake angle of diamond blade, and so on are worthy research topics, but they are beyond the scope of the proposed paper and will be saved for future investigations.

### Characterization of every second-order NLO tensor of BNA based on maker fringe measurement

Maker fringe measurement system employs a Q-switched Nd:YAG laser with a 10 ns pulse width and wavelength of 1064 nm as a pump source for the BNA crystals. By applying a notch filter to the pump beam, only SHG waves generated in BNA crystals can be detected using the silicon pin-photodiode. A half-wave plate and polarizer are installed in front of the sample and the detector, respectively, to properly control polarization states of the pump beam and the SHG beam, depending on the target element of the second-order NLO tensor. Since the principle of the Maker fringe technique is based on relative measurements of SHG intensity, a well-known standard NLO crystal is necessary. Because the magnitude of d_33_ of inorganic KTiOPO_4_ (KTP) crystal, which belong to the same point group as BNA, is well characterized with reliable accuracy^37^, it is used as a standard reference to determine absolute values of second-order NLO tensors for BNA.

**Figure 2:**
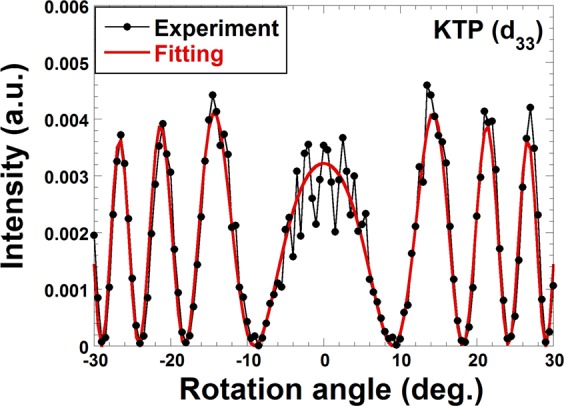
Maker fringe of d_33_ for (010)-KTP. Rotation axis of the KTP is <001>. Polarizations of pump and SHG are parallel to the rotation axis.

Figure 2 exhibits a Maker fringe pattern produced by d_33_ of Y-cut KTP crystal when the pulse energy of the pump beam is 150 μJ. The rotational axis of the KTP is its Z-axis, and the polarizations of the pump and detected SHG waves are parallel to the Z-axis. The amplitude of the fringe’s envelope represents the magnitude of d_33_, and the spacing between fringes indicates coherence length. The red line shown in Fig. 2 represents the theoretical fitting line considering anisotropy of biaxial orthorhombic crystals^32^. Here two refractive indices *n*_*z*@1064_, and n_z@532_ corresponds to the pump and SHG wavelengths as well as the amplitude coefficient *A*, containing d_33_ are fitting parameters. The thickness of the KTP crystal was measured to be 800.6 µm, which is prior given in the fitting. Meanwhile, fitted refractive indices for the pump, n_z@1064_, and SHG, n_z@532_, are 1.828 and 1.888, respectively, both of which agree well with the values derived from a given Sellmeier equation^38^. The fitted value of the amplitude coefficient, *A*, is defined to be 1, as standard reference for d_33_ = 14.7 pm/V of KTP^37^.

**Figure 3:**
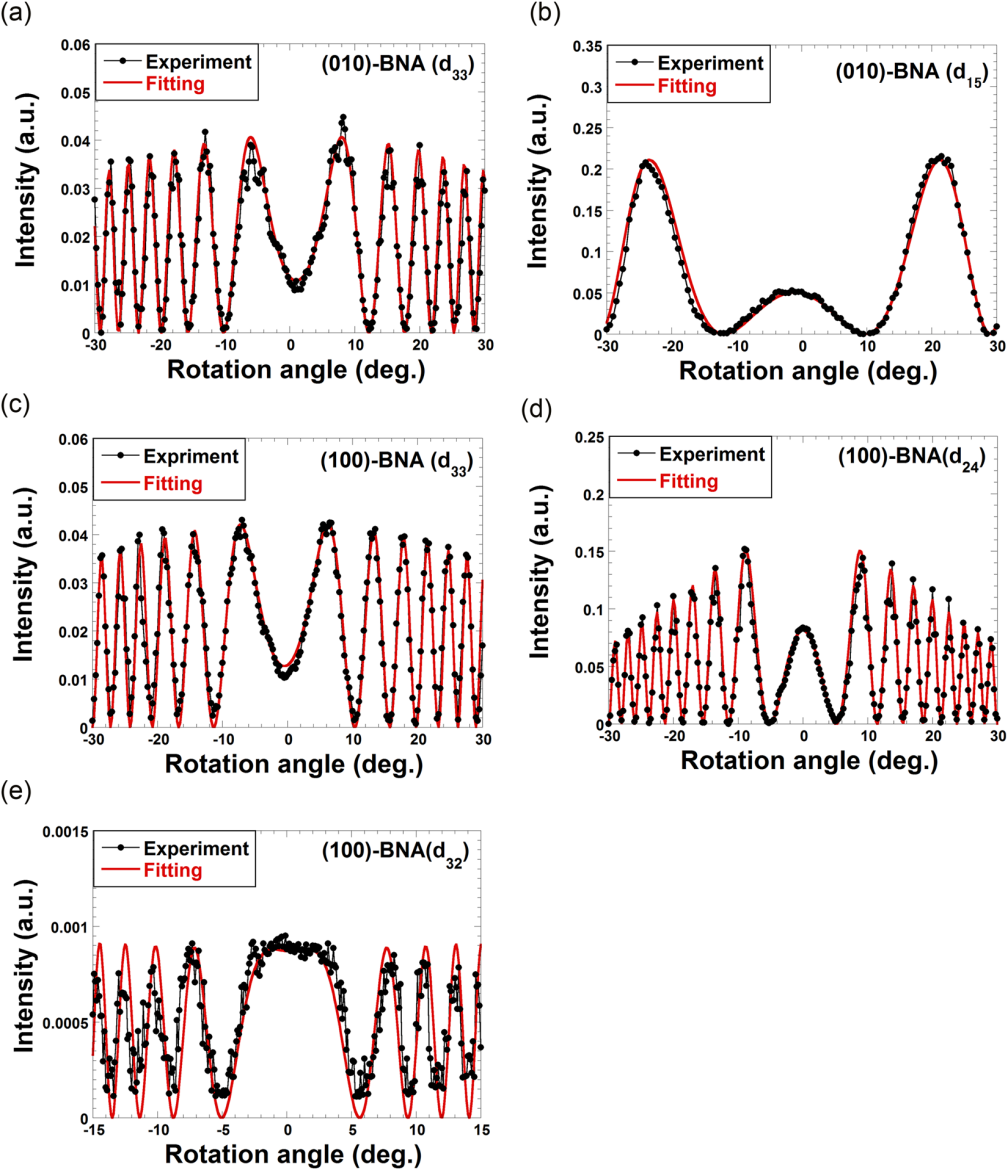
Results of Maker fringe measurements for organic BNA crystals. (**a**) Maker fringe of d_33_ for (010)-BNA slab. Rotation axis of the BNA is <001>. Polarizations of pump and SHG are parallel to the rotation axis. (**b**) Maker fringe of d_15_ for (010)-BNA slab. Rotation axis of the BNA is <100>. Polarizations of pump is tilted by 45 degree from the rotation axis. Polarization of SHG is parallel to the rotation axis. (**c**) Maker fringe of d_33_ for (100)-BNA slab. Rotation axis of the BNA is <001>. Polarizations of pump and SHG are parallel to the rotation axis. (**d**) Maker fringe of d_24_ for (100)-BNA slab. Rotation axis of the BNA is <010>. Polarizations of pump is tilted by 45 degree from the rotation axis. Polarization of SHG is parallel to the rotation axis. (**e**) Maker fringe of d_32_ for (100)-BNA slab. Rotation axis of the BNA is <010>. Polarizations of pump is parallel to the rotation axis. Polarization of SHG is perpendicular to the rotation axis.

We then measured the Maker fringe patterns for organic BNA crystals. Figure 3(a) demonstrates a result for the d_33_ element using a processed thin-parallel (010)-BNA slab. In this case, the rotational axis of the BNA is <001>, and the polarizations of both the pump and SHG are parallel to the <001> axis. The pump pulse energy of 150 μJ is maintained from the d_33_ measurement of KTP and remains fixed for subsequent all experiments. As shown in Fig. 3(a), a favorable Maker fringe pattern without distortion, asymmetry, or nonzero-minima can be successfully obtained even for organic NLO crystal when it has been precisely processed. The fitted refractive indices n_z@1064_ for the pump wavelength and n_z@532_ for the SHG wavelength are 1.802 and 2.093, respectively. The fitted amplitude coefficient, *A*, for the fringe is 15.7. By comparing the values of *A* derived from BNA and KTP, the magnitude of d_33_ of BNA can be characterized as 231 ± 5 pm/V. This value is significantly large, and its nonlinearity may be enhanced by charge transfer excitation resonance near the band-gap. Although previously reported values of d_33_ of BNA are also large^25^, our Maker fringe pattern closely traces the theoretical fitting curve. This level of similarity can only be achieved with a precisely processed plane-parallel thin BNA slab and can drastically reduce the error-bars in determining the value of NLO coefficients. Employing the same processed (010)-BNA slab and resultant Maker fringe pattern, the value of d_15_ is also characterized as shown in Fig. 3(b). In this case, the rotation axis is <100>, and the polarization of the detected SHG beam is parallel to the <100> axis. The polarization of the input pump beam is tilted 45 degrees with respect to the rotational axis <100> to generate equal pump amplitude for the <100> and <001> axes. The fitted refractive indices, n_x@1064_, n_y@1064_, n_z@1064_, for the pump wavelength, and n_x@532_ for the SHG wavelength, are 1.568, 1.707, 1.808, and 1.631, respectively. From the fitted amplitude coefficient, *A* = 5.38, the magnitude of d_15_ of BNA can be characterized as 77.6 ± 1.6 pm/V first time ever. This value is also large compared to off-diagonal elements of second-order NLO tensors of other NLO crystals and d_15_ will be useful for Type-2 phase-matched NLO processes. The first-kind Maker fringe pattern for d_31_ is also expected using the processed (010)-BNA slab; however, an explicit SHG signal cannot be observed in the experiment. This result is consistent with previous findings, which characterize d_31_ of BNA as negligibly small^25^.

To characterize the remaining elements of the second-order NLO tensor of BNA, a processed plane-parallel thin (100)-BNA slab was used. Although d_33_ of BNA was already characterized using the (010)-BNA slab, its characterization can be confirmed with the (100)-BNA slab, as exhibited in Fig. 3(c). The rotational axis of the BNA is <001>, and the polarizations of both the pump and SHG are parallel to the <001> axis in this case. A Maker fringe pattern similar to that of Fig. 3(a) can be observed because the thicknesses of the two processed slabs are almost the same. Based on fitting results for Fig. 3(c), d_33_ can be characterized as 234 ± 5 pm/V, which supports the previous result obtained using the (010)-BNA slab. In this way, same NLO coefficient can be measured for two crystals with different facets by cutting them out from a single bulk crystal for the first time.

The two remaining elements, d_24_ and d_32_, were also characterized using the (100)-BNA slab. In the case of d_24_, the rotational axis is <010>, while the detected SHG polarization is parallel to <010> and the input pump polarization is tilted 45 degrees with respect to <010>. The corresponding Maker fringe pattern is given in Fig. 3(d), and the fitted refractive indices n_x@1064_, n_y@1064_, n_z@1064_ for the pump wavelength, and n_y@532_ for the SHG wavelength are 1.538, 1.709, 1.796, and 1.784, respectively. Characterized magnitude of d_24_ is 41.4 ± 0.8 pm/V. Finally, the Maker fringe measurement for d_32_ was carried out, the result of which is given in Fig. 3(e). Herein, the rotational axis is <010>, and the polarizations of the pump and SHG are parallel and perpendicular to <010>, respectively. In this configuration, the SHG signal is relatively low and noisy compared to other Maker fringe data owing to the relatively short coherence length. The fitted refractive indices for pump wavelength, n_y@1064_, and SHG wavelengths, n_x@532_ and n_z@532_, are 1.720, 1.612 and 2.080, respectively. The magnitude of d_32_ is characterized as 78.1 ± 1.6 pm/V based on the fitting result.

The magnitudes of d_24_ and d_32_ of BNA were alternatively characterized based on the phase-matched Type-2 SHG method. These values were reported as 5.5 ± 0.2^39^ and 15.6 ± 0.9 pm/V^25^, respectively, both of which differ significantly from our results. Although the cause of these discrepancies must be clarified scientifically, we hypothesize that the Maker fringe method may produce more reliable results because the phase-matched SHG power drastically changes with even slight changes in temperature, tuning angle and so on. Another possible explanation is our improved BNA crystal quality, which likely produces higher optical nonlinearity. As for the fitted refractive indices in our Maker fringe results, small disagreements are found with the refractive indices deduced from given Sellmeier equations^25,39^. However, because multiple refractive indices, thickness of sample and magnitude of second-order NLO coefficient simultaneously influence the shape of Maker fringes, such slight discrepancy as a result of least-square fitting can be inevitable. In addition, the given Sellmeier equations obtained for BNA are not very accurate to begin with, and actual differences between the refractive indices due to crystal quality or contamination by impurities might be observed.

## Methods

**Figure 4:**
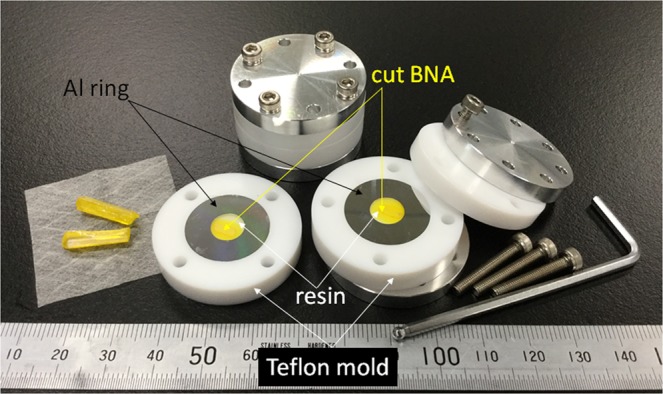
Embedded BNA bulk crystal in resin surrounded by an aluminium ring to reinforce the crystal. A teflon mold is used to flatten both surfaces and for deairing.

**Figure 5:**
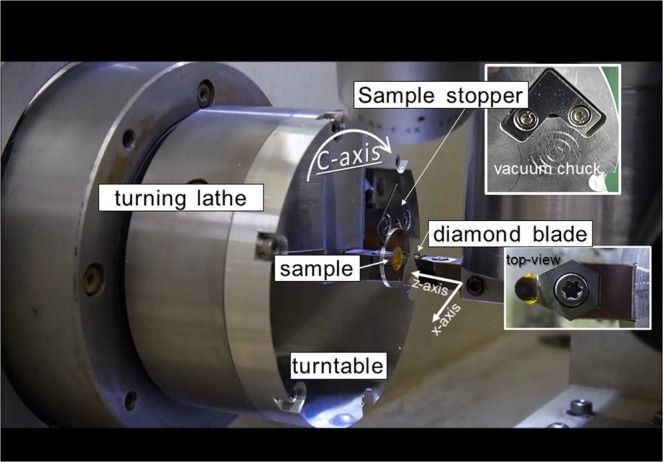
Ultra-precise prosessing machine consists of turning lathe and diamonde blade.

In general, shapes of as-grown organic crystals cannot be suitably handled for processing and fixing on the turntable of a lathe. Therefore, BNA crystals are embedded in epoxy resin and aluminum (Al) ring for easier handling and reinforcement before processing, as shown in Fig. 4. The surrounding Al ring and resin are cut together with the BNA by a diamond blade during the processing. This structure prevents cracking and distortion of the thin organic crystals. Figure 5 displays a photograph of the system. The sample is mounted on the lathe’s turntable with a vacuum chuck, and the lathe is rotated at a speed of 500 rpm. During the sample rotation, the tip of the single crystalline diamond blade contacts the surface of the sample very slowly from horizontal direction to cut the sample surface slightly. Diamond is the best blade from the viewpoints of smoothness of outline of the tip, its hardness and super thermal conductivity. During the cutting process, crystal chips are cleared from the sample surface by air blow to avoid surface damage due to scratching. To hold the sample despite friction between the sample and diamond blade, a stopper is also installed in the turning table; the height of the stopper can be controlled depending on the sample thickness. After one round-trip motion of the diamond blade along the horizontal axis, the blade approaches the sample with µm-step. This cutting process is repeated until the sample is cut to the desired thickness and then repeated for the other surface of the sample. An aerostatic bearing achieves stable rotation of the lathe without disturbing the sample, while feedback control using linear scale and precision ball-screw maintains 3D-axes scanning of the diamond blade with nm-level positioning resolution. This system enables fragile organic crystals to be processed very precisely, hence exposing any crystal facets with very smooth optical surfaces.

## Conclusion

We successfully characterized all elements of the second-order NLO tensors for organic BNA crystal for the first time by developing a method to produce thin plane-parallel (100)- and (010)-slab crystals with high-grade optical facets. Our technology enables us to not only characterize all of second-order NLO coefficients completely for any organic NLO crystals, but also fabricate new organic NLO devices for various applications.
